# Development of a Polygenic Risk Score for Metabolic Dysfunction-Associated Steatotic Liver Disease Prediction in UK Biobank

**DOI:** 10.3390/genes16010033

**Published:** 2024-12-28

**Authors:** Panagiota Giardoglou, Ioanna Gavra, Athina I. Amanatidou, Ioanna Panagiota Kalafati, Panagiotis Symianakis, Maria Kafyra, Panagiotis Moulos, George V. Dedoussis

**Affiliations:** 1 Department of Nutrition and Dietetics, School of Health Science and Education, Harokopio University, 17671 Athens, Greece; pgiardog@hua.gr (P.G.); mariakaf@hua.gr (M.K.); 2 Department of Nutrition and Dietetics, School of Physical Education, Sport Science and Dietetics, University of Thessaly, 42132 Trikala, Greece; 3 Institute for Fundamental Biomedical Research, Biomedical Sciences Research Center ‘Alexander Fleming’, 16672 Vari, Greece; moulos@fleming.gr

**Keywords:** metabolic dysfunction-associated steatotic liver disease, polygenic risk score, UK Biobank

## Abstract

Background: Metabolic dysfunction-associated steatotic liver disease (MASLD) is the leading cause of liver-related morbidity and mortality. Although the invasive liver biopsy remains the golden standard for MASLD diagnosis, Magnetic Resonance Imaging-derived Proton Density Fat Fraction (MRI-PDFF) is an accurate, non-invasive method for the assessment of treatment response. This study aimed at developing a Polygenic Risk Score (PRS) to improve MRI-PDFF prediction using UK Biobank data to assess an individual’s genetic liability to MASLD. Methods: We iteratively sequestered 10% of MRI-PDFF samples as a validation set and split the rest of each dataset into base and target partitions, containing GWAS summary statistics and raw genotype data, respectively. PRSice2 was deployed to derive PRS candidates. Based on the frequency of SNP appearances along the PRS candidates, we generated different SNP sets according to variable frequency cutoffs. By applying the PRSs to the validation set, we identified the optimal SNP set, which was then applied to a Greek nonalcoholic fatty liver disease (NAFLD) study. Results: Data from 3553 UK Biobank participants yielded 49 different SNP sets. After calculating the PRS on the validation set for every SNP set, an optimal PRS with 75 SNPs was selected (incremental R^2^ = 0.025, *p*-value = 0.00145). Interestingly, 43 SNPs were successfully mapped to MASLD-related known genes. The selected PRS could predict traits, like LDL cholesterol and diastolic blood pressure in the UK Biobank, as also disease outcome in the Greek NAFLD study. Conclusions: Our findings provide strong evidence that PRS is a powerful prediction model for MASLD, while it can also be applied on populations of different ethnicity.

## 1. Introduction

Metabolic dysfunction-associated steatotic liver disease (MASLD) is the latest term comprising a wide spectrum of liver disorders associated with metabolic syndrome, using a non-stigmatizing language [1]. MASLD is the most prevalent chronic liver disease and it has been estimated that affects approximately two billion individuals worldwide [2]. The MASLD upswing that is associated with its global prevalence of 30% requires holistic and comprehensive approach to address in-depth this public health challenge. Alongside with the manifestation of progressive hepatic damage, MASLD has been recognized as an additional risk factor for leading causes of complex diseases, such as cardiovascular disease and type 2 diabetes mellitus [3,4]. Therefore, liver-focused treatments pertain to global public health and could decrease the risk of various diseases’ progression, appreciating the complex relation between MASLD, modifiable factors, and genetic liability.

MASLD manifests hepatic steatosis in the presence of one or more cardiometabolic risk factors with low or no alcohol consumption and encompasses various liver conditions [1,5]. In some cases, simple steatosis progresses to metabolic dysfunction-associated steatohepatitis (MASH) characterized by inflammation and liver cell damage. From this stage, individuals presenting continuous inflammation and liver cell injury lead to the deposition of scar tissue (fibrosis) in the liver [6]. Consistent and advanced fibrosis can potentially progress to cirrhosis, where extensive scarring disrupts liver function and structure, increasing the risk of liver failure and the development of hepatocellular carcinoma (HCC) [7,8]. Several factors influence the onset and progression of MASLD, including genetic predisposition; environmental factors such as lifestyle (dietary habits, physical activity, and alcohol consumption) and socioeconomic factors, presence of comorbidities (e.g., obesity, and dyslipidemia), and inflammatory processes within the body [9]. Early detection, lifestyle modifications and management of comorbidities are crucial in slowing or halting the progression of MASLD.

As in other chronic disorders, the MASLD management endeavor to develop and optimize pharmacological therapies; however, lifestyle modification and weight loss remain its main cornerstone [6,8]. Due to the poor prognosis at the end-stage of the disease, early diagnosis, especially for those with advanced fibrosis enduring greater risk liver complications, is of great importance. Albeit liver biopsy is an invasive and expensive technique, it remains the gold standard for diagnosis of MASH with and without fibrosis [10]. Non-invasive alternatives for diagnosis and disease staging include scoring systems, like the Fatty Liver Index, serum biomarkers and imaging methods for hepatic stiffness. The magnetic resonance imaging proton density fat fraction (MRI-PDFF) technique has been proposed as an alternative surrogate to liver biopsy that can detect hepatic fat content in a quantitative and reproducible manner [11,12]. However, these non-invasive methods could be used for first-line screening patients with MASLD in clinical practice since they cannot fully determine long-term outcomes [13].

Since a strong heritability of MASLD susceptibility [14,15], as well as differences in prevalence and incidence by distinct race and ethnicity [16], have been supported by existing evidence, it is highly crucial to identify the genetic determinants of disease prognosis that could be useful for risk prediction. Although routine genotyping of MASLD patients is not yet recommended, the use of polygenic risk scores (PRSs), which are based on the sum of all independent risks conferred by carrying nucleotide variants, is being investigated as a useful tool to identify patients at risk of developing MASLD or related complications [17]. PRSs capture an individual’s relative risk of a disease in a specific population that naturally have a different genetic susceptibility background. Therefore, the use of PRSs would add precious value to the methodology of early disease detection, as well as effective management and treatment.

Due to this background of evidence, the aim of the present study was to generate a novel PRS to accurately predict MASLD development using data of well-characterized individuals in UK Biobank (UKBB) acquiring reported MRI-PDFF values and MASLD-related biochemical indices. After constructing a novel method in order to develop an optimum PRS (including only single-nucleotide polymorphisms |SNPs), we applied the generated PRS to a corresponding Greek non-alcoholic fatty liver Disease (NAFLD) case–control study [18] in order to validate its performance to external cohorts, as well. The goal of this study was to provide a powerful and valuable method into the quiver of MASLD clinical management in order to improve the accuracy of the prediction of future liver events as well as to provide clinicians with a tool for tailor-made treatment strategies.

## 2. Materials and Methods

### 2.1. Study Population and Phenotype

The present research analyses have been conducted using the UK Biobank Resource under Application Number 53723. Based on the information provided in Protocol 44532, the Stanford IRB has determined that the research does not involve human subjects as defined in 45 CFR 46.102(f) or 21 CFR 50.3(g). All participants of the UKBB provided written informed consent [19]. 

**Figure 1:**
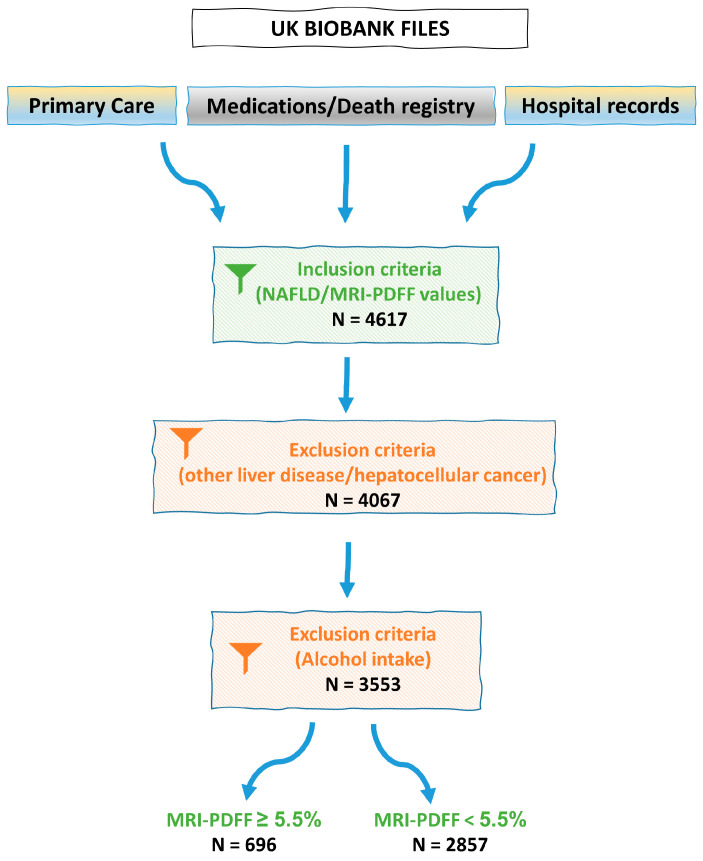
Flow chart of UKBB participants who met the inclusion/exclusion criteria for the study.

For the purpose of this study, true MASLD cases were identified by following ICD9/10 codes derived from primary care and hospital records, as also medications and death registry datasets contained in the UKBB. Patients with successful PDFF measurement assessed by abdominal MRI were included to the initial dataset and following, essential inclusion and exclusion criteria (as defined for NAFLD diagnosis at the time of analysis’ conduction, prior the new nomenclature [20]) were applied (Figure 1). Excluding individuals with a known alternative liver disease, drug-induced liver disease or excess alcohol intake (≥21 standard drinks/week for men and ≥14 standard drinks/week for women), the final dataset contained data from 3553 individuals. The outcome phenotype was based on the MRI-PDFF values, where individuals with PDFF lower than 5.5% were categorized as healthy controls; otherwise, they were stratified as individuals with hepatic steatosis.

In addition, data from the Greek NAFLD study were used. The study was approved by the Ethics Committee of Harokopio University of Athens (38074/13-07-2012), based on the Helsinki Declaration, and all participants provided a written participant consent form prior to their enrollment. Exclusion criteria of adult (>65 years) individuals were (i) presence of congenital or acquired liver disease, (ii) the exposure to hepatotoxic drugs, (iii) presence of chronic viral hepatitis, (iv) daily consumption of ethanol more than 20 g for women and more than 30 g for men, (v) the co-presence of a life-threatening disease or psychiatric disorders impairing the patient’s ability to provide written informed consent and (vi) pregnant or lactating women. After applying the aforementioned exclusion criteria, a total of 351 Caucasian individuals were recruited.

### 2.2. Genotyping Analysis

For our analysis, genotype calls derived from the UKBB study were used. Specifically, genotype calling was performed by Affymetrix on the UK BiLEVE Axiom array and the Biobank Axiom array, two closely related purpose-designed arrays and data, were directly provided by UKBB as v3 imputed data.

### 2.3. Pipeline of PRS Derivation

(i) Data filtering

The initial filter administration ensured that poorly imputed variants with IMPUTE2 INFO scores less than 0.8 were excluded from the dataset. Although no additional step regarding insertion/deletion (indels) variants was applied, only SNP variants passed this threshold. Further genotype and sample filtering was applied, using PLINK [21] software (v1.07), where SNPs with SNP call rates exceeding 5% and minor allele frequency (MAF) below 5% and samples with a sample call rate exceeding 5% were excluded from the analysis.

(ii) PRS construction and Summary statistics

An iterative PRS generation process was applied according to a similar process as previously described by Kafyra et al. 2023 [22]. For the PRS development PRSice2 [23] software package (v2.2.1) was used to perform the calculation and evaluation of PRS candidates. Initially, the quality-controlled dataset was divided into a training (90% of the total sample) and validation (10% of the total sample) datasets, using the function ‘createDataPartition’ of the caret R package [24], as it preserves variable (e.g., phenotype) properties during partitioning. The following actions concerning each iteration were performed at the training dataset. During our analysis, for each iteration, a base set (80% of the training dataset) was used to derive summary statistics, and a target set (20% of the training dataset) was used with PRSice2 along with the summary statistics to extract PRS candidates. This process was repeated 100 times, and therefore, each iteration contained a different set of base and target datasets, and as a result, 100 distinct PRSs were derived, respectively.

During the summary statistics extraction process for each iteration, principal component analysis (PCA) was applied in order to account for any underlying population stratification. Then, linear regression was performed for every base dataset (summary statistics dataset) with PLINK 1.90 to test for association between sequence variants and the MRI-PDFF value. The regression models included the covariates of sex, age, Body Mass Index (BMI), assessment center and genotype measurement batch, along with selected principal components as correction covariates, so as to assess the contribution of each SNP to the examined phenotype.

Specifically, for each iteration, the summary statistics, along with the target set, are used as inputs at the PRSice2, which then provides a specific SNP set composing the respective candidate PRS. The PRS was calculated using PRSice2 option based on the following equation:

$$\mathrm{PRS}=\sum_{i=1}^{k}\frac{\beta iGi}{N},$$

where βi represents the effect of PRS SNP i, Gi is the genotype coding (0, 1 and 2 following PLINK notation, for the number of copies of risk alleles) and N the number of samples in the population.

After the completion of the iterative PRS derivation process, SNPs that constitute the constructed PRSs were aggregated and further evaluated based on the frequency of their appearance along the PRS candidates, as some SNPs appeared only once while others more than 90% of the iterative rounds upon base/target datasets splits. Thus, according to variable frequency cutoffs of SNPs’ introduction, different SNP sets were generated.

(iii) PRS validation

For the optimal PRS construction, we applied the PRSs to the validation set. Through this step, we evaluated the derived PRSs and lessen the overfitting of our models driven by the analysis based on the training data set. We also performed permutation steps while using PRSice in application mode; specifically, PRScise performed standard analysis which was repeated after shuffling the phenotype. The procedure was then repeated for—perm 10,000, so as to perform permutation steps while also calculating limited PRSs. To evaluate the statistical significance of each PRS, we collected and calculated several performance metrics. Firstly, the *p*-value between the phenotype (MRI-PDFF) and the predicted PRS, as well as the PRS incremental R^2^ as returned by the PRSice2. Of note, PRS incremental R^2^ is the difference between R^2^ of the ‘full’ regression model that includes all the covariates/confounders and the PRS and that of the ‘basic’ regression model without the PRS. The final PRS was selected based on the best *p*-value and incremental R^2^ metrics scores.

### 2.4. SNP Analysis

SNPs of the final dataset that constitute the optimal PRS were mapped to genes using the online tool g:profiler [25], which is part of the ELIXIR infrastructure. Specifically, g:SNPsense was used to map a list of human SNP rs-codes to specific gene names. Subsequently, we used the online tool DisGENEnet [26] so as to unravel the association of genotype–disease relation of the genes retrieved from our study. Finally, for the examination of possible molecular pathways involved in the function of the genes of our interest, biomaRt R package—an interface to access Ensemble annotations—was used.

## 3. Results

### 3.1. Population Characteristics

For the purpose of our study, available data from UKBB were used. Among the participants of UKBB with genotype and phenotype data, a total of 3553 individuals were included in our analysis. Their main phenotypic characteristics are shown in Table 1. Overall, a total of 1646 (46.3%) men and 1907 (53.7%) women were included, with a median age of 57 years and a median BMI of 26.1 kg/m^2^. Moreover, 19.6% of the participants had MRI-PDFF value equal or over 5.5%, marking for MASLD disease, while 80.4% of the participants were healthy (MRI-PDFF < 5.5%), and therefore, they were classified as controls.

Regarding the genetic makeup of the UKBB participants, initial imputed data were further subjected to filtering, where variants with imputation confidence less 0.8 were excluded from the analysis. After applying quality control (QC) to filter out low-quality genotypic data, the final dataset for all downstream analysis contained 7,713,230 variants available for subsequent investigation for PRS candidates. Concerning the samples, 3499 (97.07%) exceeded the thresholds of QC filters and were included in the following analysis of the present study.

Since the main goal of the current study was to construct a novel PRS for MASLD prediction, we soughed to test whether its prediction ability applies to external populations as well. In this regard, available data from 351 individuals in a Greek case–control NAFLD study were also used during following stages of our analysis. The study consisted of adult participants free of any secondary liver disease or injury while reporting no excessive alcohol intake. Recruitment was carried out at the Outpatient Clinics of the First Department of Propaedeutic and Internal Medicine in Laiko General Hospital between 2012 and 2015. Volunteer participants were classified as cases or controls according to the level of hepatic steatosis, if any. As shown in Table 2, data from 205 women and 146 men were used, whereas the total cohort consisted of 134 cases and 217 controls. Furthermore, the predictive ‘NASH score’ [27] indicated that the study samples are mainly comprised of simple steatosis patients and not non-alcoholic steatohepatitis (NASH) patients, based on the estimated cut-off point (−1045).

### 3.2. Selection of a PRS

As thoroughly described in the Material and Methods section, following the application of QC filters, a pivotal aspect of our study involved utilizing the caret R package to partition our refined dataset into two subsets: a base dataset and a target dataset. Of note, this process was repeated 100 times in total, segmenting the data set based on the MRI-PDFF values associated with each sample. This iterative procedure of the several distinct data partitions resulted in the generation of a diverse set of 100 PRS candidates, accordingly.

Specifically, a different set of summary statistics of base dataset along with the target dataset were used as input to PRSice2 during the iterative PRS generation procedure. While indels were not explicitly excluded during the filtering process, they did not meet the statistical significance threshold for inclusion in the PRSs. Thus, each derived PRS was calculated for a different set of SNP candidates that were selected based on the PRSice2 calculations. Following, in order to develop a robust PRS, we aggregated the PRSs by creating 68 different SNP sets, each of which comprised all the SNP candidates that had passed each possible appearance threshold during the 100 iterations. In particular, the most extensive SNP set consisted of the SNPs that appeared at least once in the end of the iterative procedure, while the shortest SNP set consisted of a single SNP that appeared 90 times.

**Figure 2:**
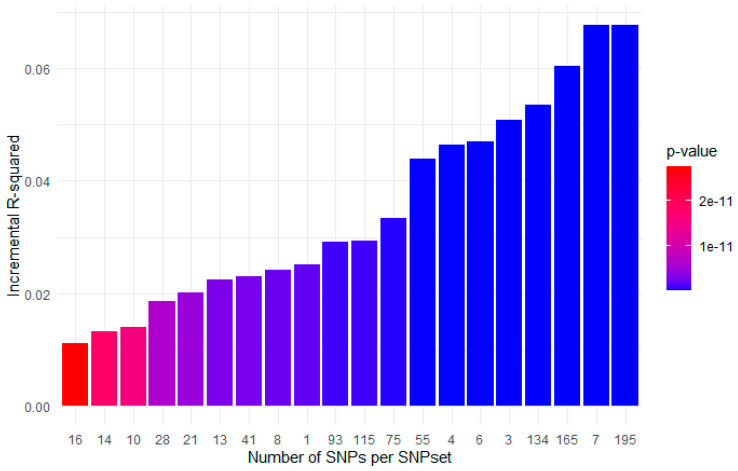
After completion of iterative and aggregation PRS derivation process, different sets of SNP contents were generated depending on frequency cutoffs. Validation of the 68 generated SNP sets resulted in 20 optimal SNP sets that were statistically significant and exhibited explanatory power for the MRI-PDFF validation dataset. X-axis: number of SNPs per SNP set; y-axis: incremental R^2^, color-coding denotes the *p*-value. For the graph illustration, ggplot2 (v 3.5.1) [28] was used.

To evaluate the performance of each of the SNP sets in composing a reliable PRS, we applied a PRS calculation through PRSice2 for every set that contained candidates derived from the aforementioned analysis to the validation dataset. Thus, regression models were fitted for each PRS against MRI-PDFF in order to evaluate their significance for the explanation of the phenotype, resulting in a series of evaluation metrics. Table 3 summarizes the 20 out of 68 derived PRSs with their corresponding SNP sets that maintain their explanatory ability towards the prediction of MRI-PDFF (incremental R-squared > 0.01) along with their respective statistical significance (*p*-value < 0.05). A corresponding graphical illustration of the 20 optimal SNP sets exhibiting high explanatory ability for the MRI-PDFF validation dataset after applying the pipeline of our analysis described above is depicted at Figure 2.

### 3.3. Evaluation of Optimal PRS

**Figure 3:**
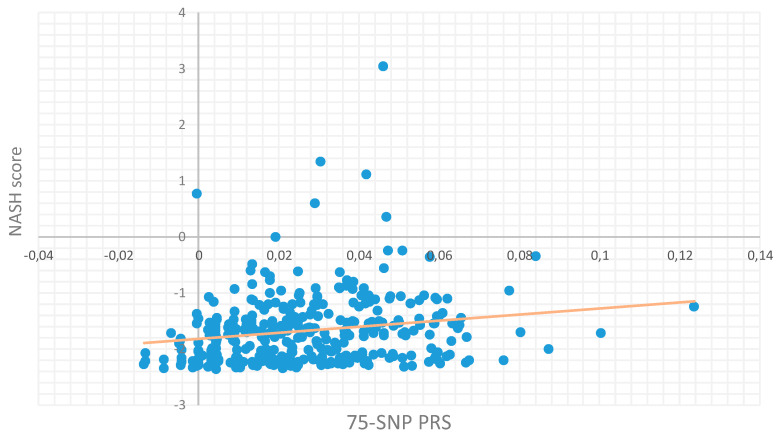
A final 75 SNPs containing PRS was selected based on high predictive value and low SNP content. The selected PRS was also evaluated for its predictive ability of NASH score in the Greek NAFLD study. The model that was generated when the 75 SNP set of the optimal PRS was applied yielded notable metrics, including *p*-value = 0.009 and incremental R^2^ value = 0.003.

In order to identify the optimal SNP set with increased predictive power, we systematically screened the 20 SNP sets obtained from the previous phase of our study. Their predictive effectiveness in terms of the NASH score in this external population was closely evaluated (Table 4), as also in terms of the case–control association (Supplementary Table S1). The aim of this analysis was the derivation of an optimal PRS that would not contain an extended number of SNPs while still exhibit equally elevated prediction value as PRSs containing higher numbers of SNPs. Therefore, the candidate PRS consisting of 75 SNPs yielding adequately high prediction power (an incremental R^2^ = 0.036) while maintaining statistical significance (*p*-value = 0.009) was selected as the optimal one. With this regard, Figure 3 shows the fit regression model of the selected PRS predicting the NASH score in the Greek NAFLD study.

**Figure 4:**
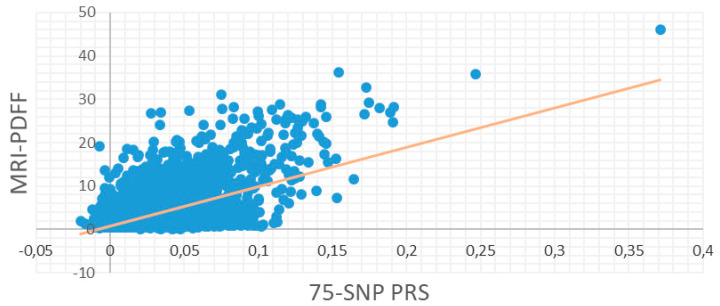
A final 75 SNPs containing PRS was selected based on high predictive value and low SNP content. The optimal PRS was applied to the whole set of UK Biobank samples and evaluated for its predicted ability of MRI-PDFF (*p*-value = 0.001 and incremental R^2^ value = 0.025).

After selecting the optimal PRS according to the metrics of the models on both the validation dataset of UKBB and the external Greek cohort, we further evaluated the selected PRS to the whole set of UKBB samples. As it was mentioned before, the effect of each SNP was the mean effect of the iterations found significant by PRSice2. In Figure 4, the fit regression model of the final PRS predicting the MRI-PDFF on the entire set of UKBB samples is presented. For this model, the *p*-value and the incremental R^2^ scored 0.001 and 0.025, respectively.

Comprehensively, the selected 75 SNPs PRS displayed a positive correlation in both aforementioned regression models, where PRS was associated with elevated MRI-PDFF and NASH score values in UKBB and the external study, respectively. Thus, the validation analyses provided strong evidence regarding the high prediction power of the PRS for MASLD derived from the UKBB upon the application of the strategy described above.

### 3.4. PRS SNPs Associated with Known MASLD-Related Traits

**Figure 5:**
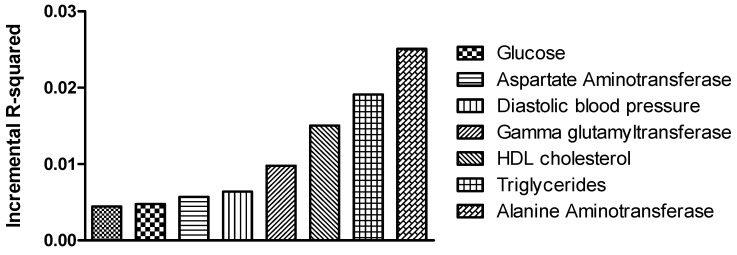
Phenotypic variance of UKBB explained for MASLD-related markers by the optimal 75 SNP-containing PRS. The graph illustration was performed using the software GraphPad Prism version 5.03.

Following, we tested to which extent the optimal PRS derived from the current study could explain the variance of classic MASLD-related biomarkers, apart from the MRI-PDFF (as implemented in R statistical language). Among the characteristics that were tested for the scope of this analysis, known markers for liver injury such as alanine aminotransferase (ALT) [29], aspartate aminotransferase (AST) and γ-glutamyltransferase (GGT) [30], along with triglycerides [31], high-density lipoprotein (HDL cholesterol) [32] and blood pressure [33], appeared to be associated with the SNP content of the optimal PRS. Figure 5 shows the reported variables that were significantly associated with the optimal PRS exhibiting adequate prediction power. The highest incremental R^2^ corresponded to the prediction of the ALT levels (incremental R^2^ = 0.018), meaning that the PRS could explain 1.8% of the variance for this trait. While most of the variance is explained by distinct predictors, such as sex, age and BMI, the PRS had an additional significant impact on the aforementioned traits improving the prediction model.

Subsequently, we sought to investigate whether given SNPs consisting of the best-performing PRS occur to respective genes associated with known MASLD-related diseases or traits. To achieve this, we initially utilized g:Profiler [34] in order to identify all the known genes that the SNPs are located on (as returned from the g:SNPense option). Upon identifying 42 genes in total (Supplementary Figure S1), we further examined their association with common MASLD-related phenotypes, using the publicly available discovery platform DisGENET [35]. Table 5 presents the gene–disease associations that were identified, suggesting that at least a portion of the 75 SNPs consisting of the optimal PRS are associated with known phenotypes, like steatohepatitis, liver cirrhosis and carcinoma, as well as diabetes, obesity and cardiovascular disorders, among others.

**Figure 6:**
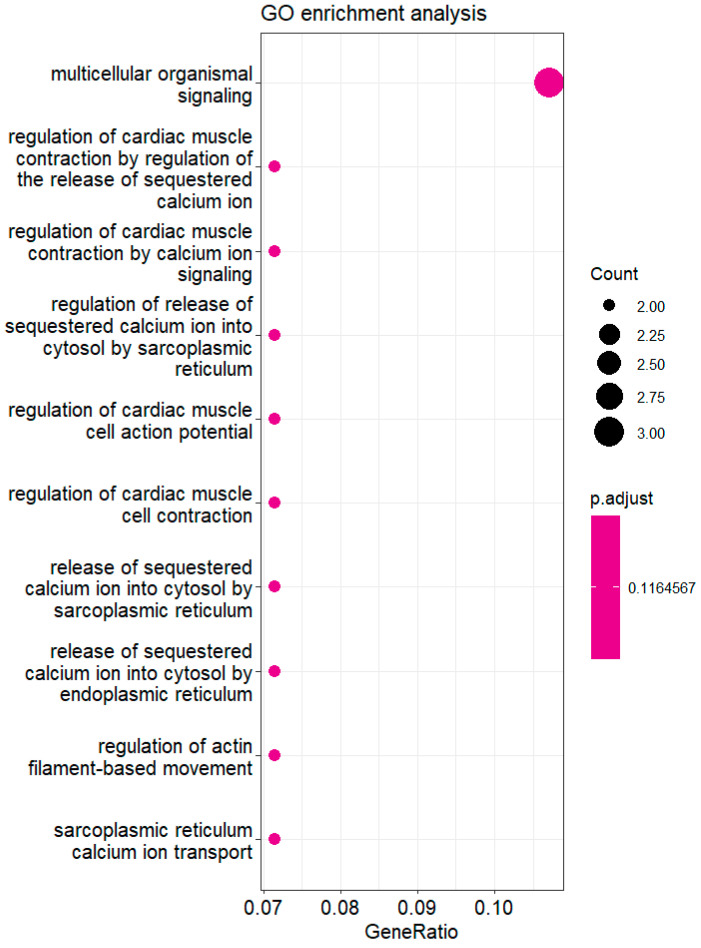
Enrichment analysis of the gene set in order to investigate molecular functions in which genes associated with the SNP set of the optimal PRS are involved.

Finally, we examined the putative molecular fingerprints in which the mapped genes are suggested to be implicated in order to further elucidate the mechanisms of action underlying the MASLD complications. The molecular pathways and the number of contributed genes using the Gene Ontology (GO) [36] tool are presented in Figure 6. Interestingly, GO analysis of the involved genes revealed that, among other molecular-level performing activities, they modulate cardiac-related pathways which needs to be further investigated. Overall, we systematically examined whether the genes involved to the optimal PRS of the current analysis were associated with MASLD and/or related phenotypes and traits. This approach intended to increase the fidelity of our findings by highlighting genes previously associated with a relevant trait that can enforced the validity of the optimal SNP set identified through our analysis.

## 4. Discussion

The goal of the present study was to employ a novel statistical analysis pipeline to construct an optimal PRS for MASLD prediction via utilizing available data from the UKBB. By employing iterative procedures, aggregating SNPs frequencies and evaluation of statistic metrics, we successfully generated a PRS containing a set of 75 SNPs, exhibiting remarkable performance on the UKBB population. Notably, the derived PRS demonstrated high applicability and effectiveness when applied to a respective, external cohort of a Greek population, overpassing ethnicity inaccuracies usually formulated by utilizing genetic data from specific populations and native backgrounds [37]. Therefore, our approach enhances the precision of the derived PRS, providing a more effective tool for predicting an individual’s risk for MASLD.

The perception of PRS is a burgeoning field emerged the last decade and has leveraged the advances of genetic tools and technologies, unveiling numerous of genetic markers linked to a broad range of complex diseases and traits [38,39,40]. From that perspective, the rise of data availability within large populations and accumulative designs has been instrumental for the increase of the predictive power of PRS. However, in order to strengthen the implementation of PRS, it is crucial to consider specific methodological designs such as the included number of SNPs [41]. In this perspective, many SNP sets exist resulting in multiple PRSs targeting the same diseases or trait. However, data derived from large multi-study consortia have shown that additional SNPs engender only minimally higher versions of the PRS supporting the idea that a specific range of SNPs can generate a relatively more stable PRS [41]. In this spectrum, we thoroughly tested the 20 best-performing PRSs derived from the UKBB study in a population with different origin (Greek study), so as to retrieve the PRS containing the optimal SNP set aiming to competent individual’s classification of risk for future MASLD-related incidences when implemented in wider practices. Therefore, it was found that the 75 SNP set PRS displayed the best statistically significant association with MRI-PDFF (incremental R^2^ value = 0.025 and *p*-value = 0.001) and Nash score (incremental R^2^ = 0.003 and *p*-value = 0.009), respectively.

Upon conducting an extensive search in the GWAS Catalog concerning the 75 polymorphisms identified to the aforementioned risk score, it has been ascertained that two employed SNPs have been previously utilized in published risk scores, namely rs3747207 and rs200210321 [42,43]. Interestingly, according to the LDlink software (v 5.6.8), the rs3747207 variant is correlated with another known variant located at the same gene, the rs738409 that has been previously associated with several types of liver disease [44]. The rs3747207 is situated on the patatin-like phospholipase domain containing 3 (*PNPLA3*) gene, representing an intron variant that has been associated with various phenotypes such as type 2 diabetes, cholesterol measurements, triglyceride, liver fibrosis, AST, hepatic cancer and MASLD. Similarly, the rs200210321 variant is mapped to the SURP And G-Patch Domain Containing 1 (*SUGP1*) gene and represents an intron variant as well. Previous research studies have established its contribution to traits including AST, liver fibrosis, cholesterol measurements, triglycerides and liver enzyme levels.

Although, there are published studies regarding non-invasive stratification of patients with MASLD-related phenotypes, such as hepatocellular carcinoma [45], this is the first attempt to generate a PRS for MASLD prediction based on the large-scale UK Biobank Data. According to the PGS (polygenic score) Catalog [46], there are 20 MASLD-related entities, including studies in alcohol-related cirrhosis [47], multi-ancestry meta-analysis [48] and multi-cancer detection liquid biopsy tests [49]. While most of these studies referred to the association or coexistence of MASLD-co-factors, like substance (tobacco and alcohol) use [50], Alzheimer’s [51] and Parkinson’s disease [52] and insulin resistance [53], our study focuses on the genetic make-up of MASLD, omitting factors such as excessive alcohol use. In the current research study, we utilized European ancestry populations (UKBB and Greek studies) in order to derive a PRS with a 75 SNP set exhibiting high prediction validity presenting comparable metrics with PRSs, including a wider number of variants.

Among the 75 SNPs composing the optimal PRS, 43 were successfully mapped to known MASLD-associated genes. Consistent with previous studies, we detected a strong association with *PNPLA3* rs3747207 polymorphism, a gene that has been independently associated with steatosis and fibrosis accumulation in various types of liver conditions [54,55]. Although it is known that the PNPLA3 protein catalyzes the hydrolysis of glycerolipids, with enzymatic activity against triglycerides, diacylglycerol and monoacylglycerol, the exact mechanism of function remains still unclear [56]. Moreover, we detected associated polymorphisms of several other genes with established contribution to both the onset and the progression of MASLD, with cAMP response element-binding protein 5 (*CREB5*), transmembrane protein with EGF-like and two follistatin-like domains 2 (*TMEFF2*), RNA Binding Motif Single Stranded Interacting Protein 3 (*RBMS3*), coiled-coil-helix-coiled-coil-helix domain containing 6 (*CHCHD6*) and retrotransposon Gag-like 1 (*RTL1*) among them. More specifically, CREB5 has been found to be overexpressed in many tumor types, including hepatocarcinoma [57,58], suggesting it is an independent prognostic factor that could be used when considered treatment strategies. With respect to the derived list of genes, Fritz and Stefanovic have also shown that RBMS3 binds to Prx1 mRNA, stabilizing its expression in fibrotic livers with a positive correlation with hepatic stellate cell activity [59]. In vivo *Chchd6* knockdown experiments in mice showed improvement of steatosis and insulin resistance as also reduced mitochondrial respiration leading to a shift toward glycolytic metabolism, thus of NAFLD, suggesting the positive correlation of the gene with MASLD progression and highlighting mitochondria dysfunction as a key mechanistic driver of the disease [60]. Moreover, Riordan et al. showed strong induction of RTL1 expression in liver tumors, suggesting that the role of its activation in the pathophysiology of hepatocarcinogenesis [61]. Albeit further research is required to fully clarify the roles, as well as the involved function mechanisms, of these genes in MASLD pathogenesis, our findings remain in accordance with the current knowledge regarding the MASLD-associated genetic loci, indicating the enhanced prediction power of the derived PRS.

Conclusively, we developed a PRS for MASLD prediction by leveraging the large sample size and comprehensive health information available in the UKBB that demonstrated high performance in both the UKBB and Greek population. Despite having achieved this goal, it is important to acknowledge certain limitations of the current study. In essence, whilst the PRS was calculated on a specific set of SNPs, additional studies utilizing larger and more diverse populations are necessary to establish a more robust PRS that can be successfully applied to a wider practice. The optimal PRS developed within this approach may be specific to the populations analyzed and may not be directly applicable to other populations with distinct genetic backgrounds or environmental contexts. Although an external Greek NAFLD was utilized for the current analysis, wider sample size would maximize the statistical power of our models. Also, further validation studies in diverse populations across different ethnicities and geographical regions are necessary to evaluate the generalizability and utility of the PRS as also to enhance its accuracy and reliability. Moreover, it is crucial to consider the potential confounding factors and limitations inherent in the genetic data utilized. While the PRS captures the genetic predisposition to MASLD, it does not encompass the entirety of the complex and multifactorial nature of the disease. Environmental factors, lifestyle choices and other non-genetic factors play important roles in the development and progression of MASLD, and their impact should not be disregarded [62]. Overall, while our research represents a significant step towards the genomic dissection of MASLD, the derived PRS requires further validation to establish its broader applicability.

In summary, our findings strongly suggest that PRS is a powerful tool for unveiling the genetic basis of complex diseases, such as MASLD, and it could potentially contribute to elevated needs and advances in personalized medicine. Although the derived PRS could improve the risk prediction additionally to traditional clinical scores such as the fibrosis 4 (FIB4) index [63], the major importance of clinical utility of PRS is concentrated to the idea of the early prediction before the onset of the disease. Therefore, the strategical pipeline described in the present study provides both a compelling polygenic prediction risk model for MASLD, as also a valuable platform for PRS methodology regarding the further development of daily clinical practice.

## 5. Conclusions

This study underscores the significant potential of PRSs towards the enhancement of the prediction and understanding of MASLD. By leveraging the comprehensive genetic data from the UKBB, we have developed and validated a PRS model that improves the predictive accuracy of MRI-PDFF for assessing MASLD. Through a novel methodological approach, we identified an optimal set of 75 SNPs that significantly contribute to the prediction of MASLD. This SNP set was determined after rigorous validation and demonstrated an incremental R^2^ of 0.025, highlighting its predictive strength. Notably, 43 of the SNPs in our optimal PRS were successfully mapped to genes already associated with MASLD, which reinforces the genetic contribution of MASLD and validates the relevance of our model. Moreover, the ability of the optimal PRS to predict MASLD outcomes, based on NASH score, in a Greek NAFLD cohort illustrates its potential applicability across different ethnic populations. This cross-population validation is crucial, as it suggests that PRS models could be implemented to predict disease risk in diverse demographic groups. However, expanding this validation across diverse populations with various origins is needed so as to further ensure its robustness and generalizability. Beyond MASLD, the PRS developed in this study also demonstrated predictive capabilities for related metabolic traits, such as LDL cholesterol levels and diastolic blood pressure. This broadens the use of the PRS model, indicating its relevance in a wider context of metabolic field. In conclusion, our findings suggest that the integration of PRS models in clinical settings could further augment the non-invasive assessment of MASLD, providing tailored risk assessments and aiding in the early detection and management of MASLD. In summary, the development of a PRS for MASLD prediction offers a promising advancement in the field of genomics and precision medicine, representing a powerful tool to improve disease prediction, enhance patient outcomes and inform targeted therapeutic strategies.

## Figures and Tables

**Table 1:** Phenotypic characteristic of participants in UK Biobank.

UK Biobank	Women (N= 1907)				Men (N = 1646)				Total (N = 3553)			
	N	Mean	Median	Range	N	Mean	Median	Range	N	Mean	Median	Range
Age (years)	-	54.94	56	40–70	-	56.53	58	40–70	-	55.68	57	40–70
BMI (kg/m^2^)	-	26.33	25.44	17.15–52.03	-	27.10	26.64	18.02–46.59	-	26.69	26.11	17.15–52.03
MRI-PDFF ≥ 5.5%	305	11.97	9.47	5.53–46.04	391	10.98	9.6	5.5–36.22	696	11.42	9.6	5.5–46.04
MRI-PDFF < 5.5%	1602	1.87	1.49	0–5.45	1255	2.2	1.92	0–5.49	2857	2.02	1.66	0–5.49

**Table 2:** Phenotypic characteristic of participants in the Greek NAFLD study.

Greek NAFLD Study	Women (N = 205)				Men (N = 146)				Total (N = 351)			
	N	Mean	Median	Range	N	Mean	Median	Range	N	Mean	Median	Range
Age (years)	-	48.33	50	20–68	-	43.59	44	18–64	-	46.35	47	18–68
BMI (kg/m^2^)	-	27.23	25.94	17.47–43.64	-	27.38	26.85	19.05–44.5	-	27.29	26.59	17.47–44.5
NASH score	-	−1.72	−1.75	−2.34–0.77	-	−1.61	−1.67	−2.36–3.04	-	−1.67	−1.72	−2.36–3.04
NAFLD cases	73	-	-	-	61	-	-	-	134	-	-	-
NAFLD controls	132	-	-	-	85	-	-	-	217	-	-	-

**Table 3:** Evaluation metrics of 20 SNP sets of PRS candidates retaining statistical significance and explanatory value when tested on the validation dataset of the UKBB. The *p*-values presented are corrected as per the PRSice2 software functionality and reporting.

SNP Set per PRS	*p*-Value	Incremental R-Squared	SNP Set per PRS	*p*-Value	Incremental R-Squared
16 SNPs	2.91 × 10^−16^	0.0677187	115 SNPs	7.84 × 10^−13^	0.0291848
14 SNPs	2.93 × 10^−16^	0.0676814	75 SNPs	1.72 × 10^−12^	0.0252194
10 SNPs	1.37 × 10^−15^	0.0603309	55 SNPs	2.10 × 10^−12^	0.0242348
28 SNPs	5.69 × 10^−15^	0.0534521	4 SNPs	2.67 × 10^−12^	0.0230095
21 SNPs	9.96 × 10^−15^	0.0507356	6 SNPs	2.96 × 10^−12^	0.0224874
13 SNPs	2.17 × 10^−14^	0.0469401	3 SNPs	4.62 × 10^−12^	0.020233
41 SNPs	2.46 × 10^−14^	0.0463197	134 SNPs	6.26 × 10^−12^	0.0186896
8 SNPs	4.00 × 10^−14^	0.0439395	165 SNPs	1.53 × 10^−11^	0.0141207
1 SNPs	3.44 × 10^−13^	0.0332965	7 SNPs	1.81 × 10^−11^	0.013285
93 SNPs	7.68 × 10^−13^	0.0292876	195 SNPs	2.76 × 10^−11^	0.0110975

**Table 4:** Evaluation metrics of 20 SNP sets of PRS candidates when the association with NASH score on Greek NAFLD study was tested.

SNP Set per PRS	*p*-Value	Incremental R-Squared	SNP Set per PRS	*p*-Value	Incremental R-Squared
165 SNPs	0.410	0.009172473	16 SNPs	2.54 × 10^−8^	0.112329061
195 SNPs	0.386	0.009688416	14 SNPs	3.61 × 10^−10^	0.135347290
134 SNPs	0.281	0.012364314	13 SNPs	1.16 × 10^−11^	0.153375274
115 SNPs	0.218	0.014383719	6 SNPs	1.54 × 10^−12^	0.163799510
93 SNPs	0.056	0.024421329	7 SNPs	1.54 × 10^−12^	0.163799510
75 SNPs	0.009	0.036591760	8 SNPs	1.54 × 10^−12^	0.163799510
55 SNPs	0.003	0.042302079	10 SNPs	3.52 × 10^−14^	0.182859128
41 SNPs	0.000	0.058940611	3 SNPs	3.49 × 10^−22^	0.269104706
28 SNPs	6.50 × 10^−7^	0.09421087	4 SNPs	3.49 × 10^−22^	0.269104706
21 SNPs	4.05 × 10^−7^	0.0968916481	1 SNPs	1.80 × 10^−34^	0.383239846

**Table 5:** Investigation of genes where optimal PRS-derived SNPs are located unraveled associations with common MASLD-related diseases or traits.

Disease Trait	Gene
MASLD	*pnpla3, creb5, tmeff2, rbms3, chchd, rtl1*
Diabetes	*sugp1, tmeff2, pnpla3, vwf, thrb, ptprt, fbln5*
LDL cholesterol	*sugp1, lims1, cntnap2, spry4-as1*
Obesity	*pnpla3, vwf, sugp1, thrb, rtl1, ank2, gna13, slc4a4*
Triglycerides	*sugp1, thrb, vwf, pnpla3*
Hyperthyroidism	*vwf, thrb*
Liver cirrhosis	*pnpla3, vwf, cntnap2, rbms3*
Diastolic blood pressure	*ank2, fbln5, rbms3, sugp1*
Systolic blood pressure	*spry4-as1, slc28a1, creb5, sugp1*
Steatohepatitis	*pnpla3, rtl1, thrb, sugp1, deptor*
Cardiovascular diseases	*vwf, pnpla3, ank2, fbln5. dab2ip*
Liver carcinoma	*csmd1, pnpla3, dab2ip, vwf, rbms3, sugp1, tincr, fbln5, ccdc178, atg10, deptor, thrb, ptprt, gna13, creb5, slc28a1, rtl1*
Serum total cholesterol	*sugp1, pnpla3, lims1, spry4-as1*

## Data Availability

This research was conducted using the UK Biobank Resource under Application Number 53723. Requests to access the data should be made via application to UK Biobank. Greek NAFLD participants’ data are not publicly available due to participants’ privacy and ethical restrictions.

## Supplementary Materials

The following supporting information can be downloaded at: https://www.mdpi.com/article/10.3390/genes16010033/s1, Table S1: Evaluation metrics of 20 SNP-sets of PRS candidates when tested for case-control association on the Greek NAFLD study population; Figure S1: Association of SNPs contained in the selected PRS with 42 known genes (for data presentation, g:Profiler was used).
