# Supplementary material for: Development of a Polygenic Risk Score for Metabolic Dysfunction-Associated Steatotic Liver Disease Prediction in UK Biobank
Source: Genes (Basel). 2024 Dec 28;16(1):33. doi: 10.3390/genes16010033 (PMC11765347; doi:10.3390/genes16010033)
Supplement: Supplementary file 1 [file genes-16-00033-s001.zip › genes-3356184-supplementary.pdf]

Supplementary material

Supplementary Table S1: Evaluation metrics of 20 SNP-sets of PRS candidates when tested for case-control association on the Greek NAFLD study population.

**SNP set per PRS**	***p*-value**
1 SNPs	0.016125
3 SNPs	0.175119
4 SNPs	0.175119
6 SNPs	0.263056
7 SNPs	0.263056
8 SNPs	0.263056
10 SNPs	0.269239
13 SNPs	0.223698
14 SNPs	0.220916
16 SNPs	0.160556
21 SNPs	0.16355
28 SNPs	0.171232
41 SNPs	0.732674
55 SNPs	0.378025
75 SNPs	0.03465
93 SNPs	0.101555
115 SNPs	0.222508
134 SNPs	0.21817
165 SNPs	0.118898
195 SNPs	0.05462

Supplementary Figure S1: Association of SNPs contained in the selected PRS with 42 known genes (for data presentation, g:Profiler was used).

id	chr	start	end	strand	gene ids	gene names ↓	3_prime_UTR_variant	NMD_transcript_variant	intron_variant	non_coding_transcript_exon_variant	non coding transcript variant
rs11064034	12	6120555	6120555	+	ENSG00000110799	VWF		2	8		2
rs12693641	2	192175913	192175913	+	ENSG00000144339	TMEFF2			4		
rs191197547	19	5577294	5577294	+	ENSG00000223573	TINCR			2		2
rs111227523	3	24491429	24491429	+	ENSG00000151090	THRB		1	16		
rs114300125	5	168088199	168088199	+	ENSG00000145934, E...	TENM2, ENSG000002...		1	10		3
rs200210321	19	19283081	19283081	+	ENSG00000105705	SUGP1		3	5		1
rs79338401	5	142439711	142439711	+	ENSG00000231185	SPRY4-AS1			5		5
rs183525565	4	71347873	71347873	+	ENSG00000080493	SLC4A4			9		1
rs147980895	15	84930657	84930657	+	ENSG00000156222	SLC28A1			6		
rs141010401	17	268901	268901	+	ENSG00000181031	RPH3AL			11		2
rs73049025	3	29944891	29944891	+	ENSG00000144642	RBMS3		1	9		1
rs4812661	20	42946483	42946483	+	ENSG00000196090	PTPRT		3	27		3
rs3747207	22	43928975	43928975	+	ENSG00000100344	PNPLA3		3	12		3
rs145876990	14	100903253	100903253	+	ENSG00000225746, E...	MEG8, RTL1, MIR493HG			13		11
rs117782433	12	90650358	90650358	+	ENSG00000286021, E...	LINC02822, ENSG000...			9		9
rs562709575	4	13801259	13801259	+	ENSG00000250634, E...	LINC01182, ENSG000...			8		8
rs72833158	2	108566448	108566448	+	ENSG00000169756	LIMS1			3		
rs112581770	16	84667191	84667191	+	ENSG00000135686	KLHL36	2			2	
rs78317030	17	65037967	65037967	+	ENSG00000120063	GNA13			2		
rs200956457	9	37742906	37742911	+	ENSG00000070601, E...	FRMPD1, ENSG00000...		2	6		
rs561368644	14	91873894	91873894	+	ENSG00000140092	FBLN5		3	14	1	3
rs62337379	4	110944678	110944678	+	ENSG00000288692	ENSG00000288692			1		1
rs111368831	2	138931471	138931471	+	ENSG00000287147	ENSG00000287147			1		1
rs190264911	19	40063065	40063065	+	ENSG00000269749	ENSG00000269749			1		1
rs138729606	12	19989707	19989707	+	ENSG00000255910	ENSG00000255910			6		6
rs188764105	5	162510223	162510223	+	ENSG00000254186	ENSG00000254186			2		2
rs150094120	8	32945378	32945378	+	ENSG00000247134	ENSG00000247134			12		12
rs145721802	20	36052593	36052593	+	ENSG00000234139	ENSG00000234139			1		1
rs149283822	8	119920622	119920622	+	ENSG00000155792	DEPTOR			2		
rs202155207	9	121781405	121781405	+	ENSG00000136848	DAB2IP			4		
rs117465983	8	3688531	3688531	+	ENSG00000183117	CSMD1			5		
rs149552869	7	28746159	28746159	+	ENSG00000146592	CREB5			8		2
rs80143958	7	147722006	147722006	+	ENSG00000174469	CNTNAP2			5		4
rs115800252	3	126904420	126904420	+	ENSG00000159685	CHCHD6		1	6		2
rs185025747	18	33423531	33423531	+	ENSG00000166960	CCDC178			8		
rs113594646	4	113435912	113435912	+	ENSG00000145349	CAMK2D		1	1		
rs115304623	4	86903587	86903587	+	ENSG00000285458	C4orf36			2		1
rs190953742	10	126644202	126644202	+	ENSG00000154493	C10orf90			2		1
rs76070271	12	100094720	100094720	+	ENSG00000111647	BLTP3B			2		
rs80220222	5	82275941	82275941	+	ENSG00000152348, E...	ATG10, RPS23	4		4		2
rs61267165	4	112947610	112947612	+	ENSG00000145362	ANK2		1	58		7
rs114637914	4	174854767	174854767	+	ENSG00000168594	ADAM29			4		4
rs112021791											
rs113845636											
rs115513607											
rs116046204											
rs116865008											
rs118167207											
rs13136502											
rs139270183											
rs142464902											
rs144162749											
rs144582390											
rs146237189											
rs148002590											
rs148712755											
rs149962371											
rs183322630											
rs190090544											
rs67167275											
rs6870905											
rs72836222											
rs72998516											
rs73241051											
rs7544434											
rs76271662											
rs77490847											
rs78401030											
rs78404125											
rs79023015											
